# Role of Chromosome- and/or Plasmid-Located *bla*_NDM_ on the Carbapenem Resistance and the Gene Stability in Escherichia coli

**DOI:** 10.1128/spectrum.00587-22

**Published:** 2022-08-02

**Authors:** Noriko Sakamoto, Yukihiro Akeda, Yo Sugawara, Yuki Matsumoto, Daisuke Motooka, Tetsuya Iida, Shigeyuki Hamada

**Affiliations:** a Japan-Thailand Research Collaboration Center for Infectious Diseases, Research Institute for Microbial Diseases, Osaka University, Osaka, Japan; b Antimicrobial Resistance Research Center, National Institute of Infectious Diseases, Tokyo, Japan; c Department of Bacteriology I, National Institute of Infectious Diseases, Tokyo, Japan; d Department of Infection Metagenomics, Genome Information Center, Research Institute for Microbial Diseases, Osaka University, Osaka, Japan; e Center for Infectious Disease Education and Research, Osaka University, Japan; University at Albany, State University of New York

**Keywords:** carbapenemase-producing *Enterobacterales*, *E. coli*, carbapenem, *bla*_NDM_, β-lactamase

## Abstract

The spread of New Delhi metallo-β-lactamase (NDM)-producing *Enterobacterales* represents a public health risk. The horizontal transfer of plasmids encoding the NDM gene, *bla*_NDM_, usually mediates its spread to other bacteria within the family. In contrast, *Enterobacterales* with a chromosome-located *bla*_NDM_ is rarely reported. The phenotypic differences between chromosome- and plasmid-located carbapenemase genes are poorly understood. To determine the significance in terms of the location of drug resistance genes, we examined carbapenemase activity and stability of chromosome- and plasmid-located *bla*_NDM_. Escherichia coli M719 possessing both chromosomes- and plasmid-located *bla*_NDM_ genes was used as a wild-type strain (WT) for the construction of mutants, Δp*bla*_NDM_ and Δc*bla*_NDM_, wherein chromosome- or plasmid-located *bla*_NDM,_ was knocked out, respectively. The mutant Δp*bla*_NDM_ showed lower hydrolyzing activity against imipenem and gene expression than the WT or Δc*bla*_NDM_ mutant. The MICs of both mutant strains were still above the breakpoint of imipenem and meropenem. Moreover, the chromosome-located *bla*_NDM_ gene was stable for at least 30 days in the absence of antimicrobial pressure, whereas the Δc*bla*_NDM_ mutant lost *bla*_NDM_ to 87% at 30 days compared to that of the initial inoculum. Organisms harboring the plasmid-located carbapenemase genes were found to provide a higher level of carbapenem resistance than those with chromosome-located genes. However, the latter organisms with chromosomal carbapenemase genes exhibited more stable carbapenem resistance than did the former ones. In summary, chromosomally located carbapenemase genes require further monitoring and more attention should be paid to them.

**IMPORTANCE** Carbapenem-resistant *Enterobacterales* (CRE) carrying *bla*_NDM_ have spread worldwide since they were first reported in 2009. Many studies using whole-genome sequencing have identified the genetic structures, plasmid scaffolds of *bla*_NDM_, and mechanisms of spread via horizontal transfer. Chromosome-located *bla*_NDM_ and integration mechanisms from plasmids have rarely been reported, and their significance is not fully understood. Here, we showed that the chromosome-located *bla*_NDM_ was associated with lower levels of carbapenem resistance and carbapenemase activity than the plasmid-located *bla*_NDM_. However, it conferred carbapenem resistance above the breakpoints and the loss of chromosome-located *bla*_NDM_ was not observed in the absence of antibiotic pressure. This study suggests that CRE strains carrying chromosome-located *bla*_NDM_ may persist in clinical and environmental settings for a long period even without antibiotic pressure and need to be monitored along with plasmid-located *bla*_NDM_.

## OBSERVATION

Carbapenem-resistant *Enterobacterales* (CRE) represents a major health concern in clinical settings. Various types of carbapenemases have been identified, including NDM, IMP, VIM, KPC, and OXA-48 type. NDM-type carbapenemase originating from the Indian subcontinent has rapidly spread to different parts of the world, including Southeast Asia (1–5). The NDM gene, *bla*_NDM_, is typically located in plasmids that facilitate the spread of carbapenem resistance via horizontal gene transfer. Plasmids act as scaffolds that assemble arrays of antibiotic resistance genes, thereby generating multiple-drug-resistant phenotypes in members of the order *Enterobacterales*. However, few CREs have been reported to carry the *bla*_NDM_ on their chromosome (6–9). Although the genetic contexts and integration mechanisms of chromosome-located carbapenemase genes have been reported occasionally, the difference in phenotype between isolates with the chromosome- and plasmid-located genes has not been extensively studied. Mathers et al. (10) identified five K. pneumoniae clinical isolates showing chromosomal integration of *bla*_KPC_. They compared the MICs of meropenem and ertapenem between these isolates and others harboring plasmid with *bla*_KPC_. They found that chromosomal genes exhibited weaker carbapenemase activity and lower expression of *bla*_KPC_. However, the genetic background, including sequence types (STs) and porin gene status, is variable among individual isolates, and the prediction of an accurate correlation between carbapenem resistance and location of the carbapenemase gene is challenging. Therefore, phenotypic assays using the same genetic background are required to evaluate the true significance in terms of carbapenem resistance between chromosomal or plasmid-located carbapenemase genes. Here, we isolated an E. coli strain possessing *bla*_NDM-5_ on plasmid as well as chromosome and used it in further experiments to explore the comparative significance of chromosome- and plasmid-located *bla*_NDM_ in CRE.

**FIG 1:**
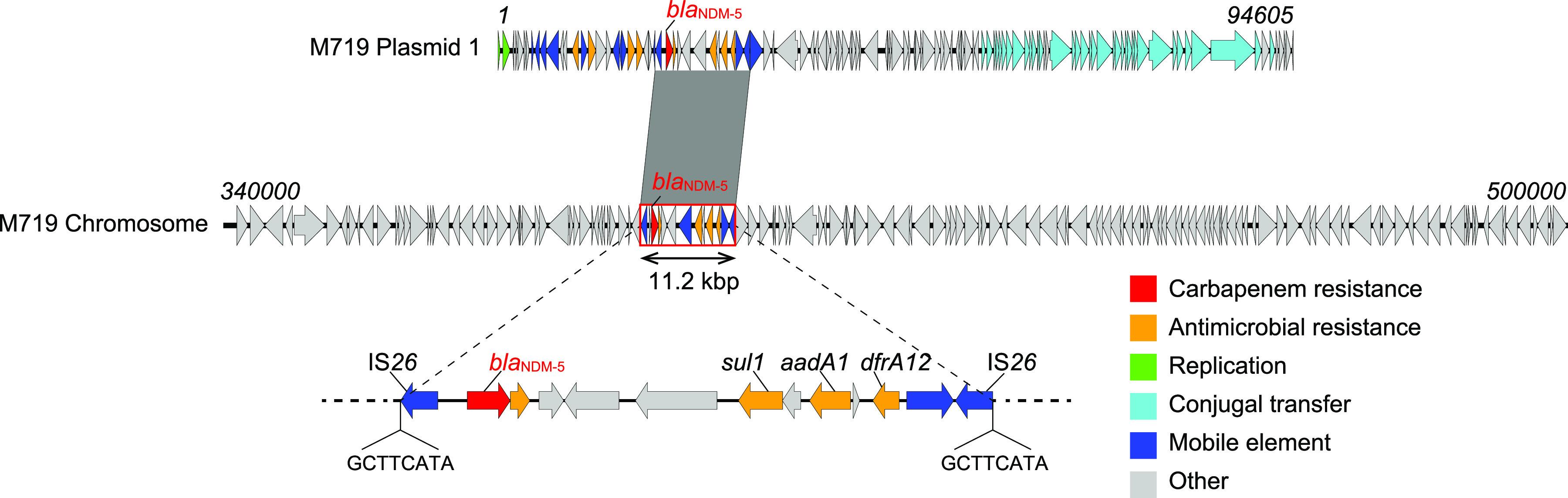
Comparison of the chromosomal region (11.2 kbp) containing *bla*_NDM-5_ in E. coli M719 and the *bla*_NDM-5_-located region in the pM71901 genome. The area shaded in dark gray indicates the region possessing the identical DNA sequence (with > 99% similarity) between the two genomes. The 11.2 kbp region containing *bla*_NDM-5_ is highlighted in a red box. Nucleotide letters below the sequences of the M719 chromosome represent target site duplications.

Among CRE isolates previously obtained from CRE surveillance at a tertiary-care hospital in Yangon, Myanmar (3–5), E. coli isolate M719 carrying *bla*_NDM_ on the plasmid and chromosome was found using S1 nuclease pulsed-field gel electrophoresis (S1-PFGE) combined with Southern hybridization targeting *bla*_NDM_. This isolate belonged to ST8453 which was originally assigned to a clinical isolate from Yangon (5). Whole-genome sequencing using the MinION system on E. coli isolate M719 identified multiple resistance genes on the chromosome and plasmid genomes (Table S1 in Supplemental File 1). The chromosome of M719 was found to include an 11.2 kbp region that contained *bla*_NDM-5_ and three other antimicrobial resistance genes against aminoglycosides, sulfonamide, and trimethoprim, which were bracketed by IS*26*. This region was integrated between a quinone oxidoreductase gene and the *dna*B gene and shared > 99% sequence identity with that integrated into the IncFII *bla*_NDM-5_-encoding plasmid (94.6 kbp) of M719 (Fig. 1). We also found 8 bp direct repeats adjacent to the IS*26* copies, indicating intermolecular transposition from pM71901 to the chromosome (Fig. 1). IS*26* is commonly detected in plasmids in *Enterobacterales* and surrounding regions of the chromosome and *bla*_NDM_ (7, 11). The chromosomal integration of drug-resistant genes and virulence genes via IS*26* has frequently been reported recently (12, 13). Therefore, chromosomal integration of *bla*_NDM_ via IS*26*-mediated transposition and/or homologous recombination from plasmids are likely to occur in various CRE organisms.

**FIG 2:**
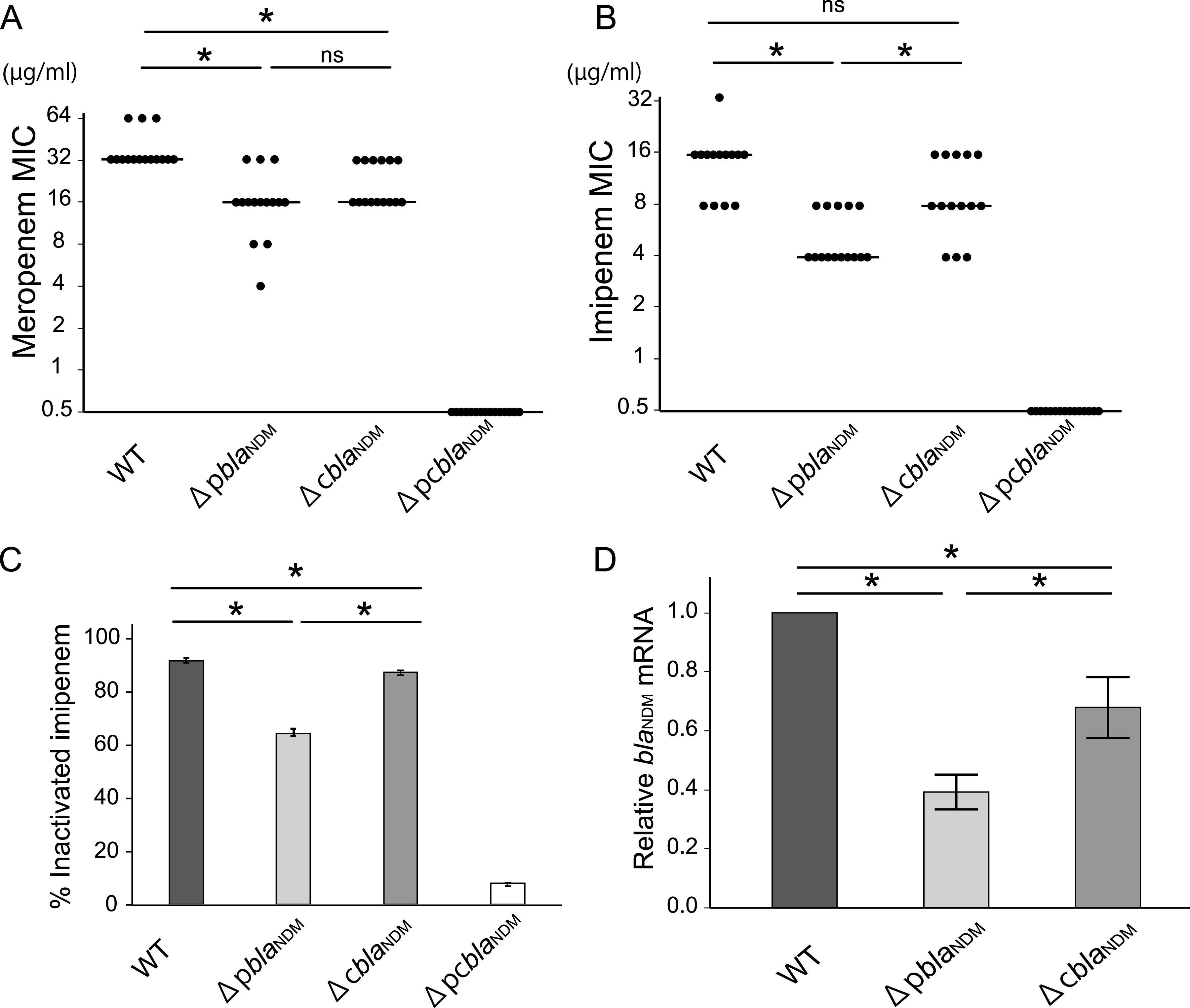
Comparative features of E. coli M719 and its *bla*_NDM_ mutants in terms of MIC to meropenem/imipenem, inactivation of imipenem, and *bla*_NDM_ mRNA levels in their cultures. MICs of (A) meropenem and (B) imipenem stratified based on the location of *bla*_NDM_ in Escherichia coli M719. Solid lines represent median MICs. The significance of *P* values was determined using the Steel-Dwass test (*, *P* < 0.05). (C) Hydrolysis of imipenem by the WT and knockout mutants (Δp*bla*_NDM_ and Δc*bla*_NDM_) after 30 min incubation. Statistical comparisons were performed using the Steel-Dwass test for multiple comparisons. *, *P* < 0.05; ns, not significant. Error bars represent standard deviation. (D) Relative mRNA levels of *bla*_NDM-5_ as determined with qPCR. Values were normalized to the mRNA level in the WT. Statistical comparisons were performed using the Steel-Dwass test for multiple comparisons; *, *P* < 0.05. Error bars represent standard deviations.

We used M719 (WT) harboring one copy of the *bla*_NDM-5_ gene in its chromosome and plasmid for the construction of mutants by deleting the plasmid- or chromosomal-located *bla*_NDM-5_ gene, yielding a plasmid-located *bla*_NDM-5_ knockout mutant (Δp*bla*_NDM-5_) and chromosomal *bla*_NDM-5_ knockout mutant (Δc*bla*_NDM-5_) (Fig. S1 in Supplemental File 1). The MICs of imipenem and meropenem against mutants Δp*bla*_NDM-5_ and Δc*bla*_NDM-5_ were lower than those against the WT strain (Fig. 2A). Imipenem showed a lower MIC value against mutant Δp*bla*_NDM-5_ than against mutant Δc*bla*_NDM-5_ (*P* < 0.05), while significant difference was not observed between meropenem MIC values against the two mutants. This observation could be related to the difference in permeability of these two beta-lactams (14), although the detailed mechanism is unclear. Consistent with the above result, the hydrolytic activity of imipenem by Δp*bla*_NDM-5_ was significantly lower than that of the WT and Δc*bla*_NDM-5_ mutant (*P* < 0.05) (Fig. 2B and C). The hydrolytic activity of Δp*bla*_NDM-5_ mutant also exhibited a significant decrease, compared to that of the WT. The double-knockout mutant, Δpc*bla*_NDM-5_ was also constructed, and it was found to be susceptible to both carbapenems and showed no hydrolytic activity against imipenem. A recent study demonstrated that the permeability of meropenem was substantially lower than imipenem in K. pneumoniae and E. cloacae (14). Therefore, meropenem penetrated periplasm could be hydrolyzed immediately by a certain amount of the NDM enzyme, resulting in a higher MIC value. As a result, our meropenem MIC assay may have not shown a significant difference between the Δp*bla*_NDM-5_ and Δc*bla*_NDM-5_ mutants. These results indicated that organisms carrying plasmid-located *bla*_NDM_ exhibited higher levels of carbapenem resistance and enhanced carbapenemase activity than mutants carrying chromosomal *bla*_NDM_ only. A similar phenomenon has been reported in clinical isolates carrying chromosomal *bla*_KPC_, which exhibited lower MIC and tested negative or weakly positive in phenotypic tests probably due to a single chromosomal copy number with a lower mRNA level (10). We then investigated the correlation between the copy number of the carbapenemase gene and the mRNA level of the carbapenemase gene and the MICs against carbapenems (15–17). The WT containing two copies of *bla*_NDM-5_ (chromosome- and plasmid-located) showed the highest level of carbapenem resistance and *bla*_NDM-5_ mRNA level. The transcription levels of *bla*_NDM-5_ in mutant Δp*bla*_NDM-5_ and Δc*bla*_NDM-5_ were 0.4-fold and 0.7-fold compared to those of the WT, respectively (Fig. 2D). These results were consistent with those of MICs of carbapenems and hydrolytic activity against imipenem (Fig. 2C), showing that the mRNA level of *bla*_NDM-5_ and carbapenemase activity are positively correlated. We also analyzed the sequences of the promoter region upstream of chromosomal and plasmid-mediated *bla*_NDM-5_. We found that excluding promoter sequences involved in the difference in transcription, the 51 bp adjacent sequence of *bla*_NDM-5_ was completely conserved and identical to the promoter region of *bla*_NDM-1_ as described previously (Fig. S2 in Supplemental File 1) (18). The *bla*_NDM-5_ mRNA levels in mutant Δc*bla*_NDM-5_ were 1.73-fold higher than those of mutant Δp*bla*_NDM-5_, suggesting that the former harboring plasmid with *bla*_NDM-5_ showed higher levels of *bla*_NDM-5_ transcription and carbapenem resistance than did the latter with chromosomal *bla*_NDM-5_. The increase in the mRNA level of Δc*bla*_NDM-5_ likely reflects the plasmid copy number of pM71901, and we subsequently estimated the plasmid copy number of the *bla*_NDM-5_ gene of pM71901 in the WT strain. The plasmid copy number of pM71901 was 2.12, which is similar to the copy number of IncFII plasmids, which are recognized as low-copy-number plasmids.

*In vitro* and *in silico* experiments have demonstrated that plasmid-located drug resistance genes are frequently lost from plasmids under antibiotic-free conditions, i.e., deprived selection pressures (19–21). Therefore, we tested the stability of *bla*_NDM_ in the absence of carbapenems. During the 30 days of daily passaging in the absence of meropenem, mutant Δp*bla*_NDM-5_ retained chromosomal *bla*_NDM-5_, while mutant Δc*bla*_NDM-5_ showed a gradual loss of the plasmid-located *bla*_NDM-5_ with a 13% reduction in the population (Table S2 in Supplemental File 1). In contrast, the loss of *bla*_NDM-5_ was not observed in the serially passaged culture in the presence of meropenem. These results suggested that the chromosome-located *bla*_NDM-5_ was stable in the absence of selective pressure of antibiotics.

In conclusion, we demonstrated unique features of plasmid/chromosome-located *bla*_NDM-5_. The plasmid-carrying *bla*_NDM-5_ exhibited higher levels of hydrolyzing activity and resistance against imipenem than did the chromosome-located gene because of its higher copy number, and frequent transmission to other bacteria via conjugation. Our results could apply to other antimicrobial resistant determinants that can be found on chromosomes and plasmids. However, it might not apply even to the same gene because the copy number depends on plasmid types and transcriptional activity should be affected by other factors than plasmid copy number. It should not also apply to the case in which a single copy of an antimicrobial-resistant determinant is sufficient to confer resistance on its bacterial host. Meanwhile, it is interesting to explore an upper threshold of β-lactamase genes’ copy number at which there is not an increase in the relative degradation of substrates of their encoding enzymes. This could be plausible because they are usually located in the periplasmic space or, for NDM-type β-lactamases, anchored to the outer membrane (22), which could potentially reach a saturation.

Although chromosomal *bla*_NDM-5_ showed essentially no conjugation ability and a relatively lower level of carbapenem resistance, it was stably carried in CRE even in the absence of antimicrobial selective pressure. The stability of *bla*_NDM_ via chromosomal integration in outbreak clones or environmental isolates may pose a serious public health concern. Further studies are required to understand the biological behavior of CREs with chromosomal carbapenemase genes, which may be of epidemiological significance.

## References

[1] Yong D, Toleman MA, Giske CG, Cho HS, Sundman K, Lee K, Walsh TR. 2009. Characterization of a new metallo-β-Lactamase Gene, *bla*_NDM-1_, and a novel erythromycin esterase gene carried on a unique genetic structure in *Klebsiella pneumoniae* sequence type 14 from India. Antimicrob Agents Chemother 53:5046–5054. doi:10.1128/AAC.00774-09. 19770275 PMC2786356

[2] Rimrang B, Chanawong A, Lulitanond A, Wilailuckana C, Charoensri N, Sribenjalux P, Phumsrikaew W, Wonglakorn L, Kerdsin A, Chetchotisakd P. 2012. Emergence of NDM-1- and IMP-14a-producing *Enterobacteriaceae* in Thailand. J Antimicrob Chemother 67:2626–2630. doi:10.1093/jac/dks267. 22796889

[3] Sugawara Y, Akeda Y, Sakamoto N, Takeuchi D, Motooka D, Nakamura S, Hagiya H, Yamamoto N, Nishi I, Yoshida H, Okada K, Zin KN, Aye MM, Tomono K, Hamada S. 2017. Genetic characterization of *bla*_NDM_-harboring plasmids in carbapenem-resistant *Escherichia coli* from Myanmar. PLoS One 12:e0184720. doi:10.1371/journal.pone.0184720. 28910381 PMC5598989

[4] Sugawara Y, Akeda Y, Hagiya H, Sakamoto N, Takeuchi D, Shanmugakani RK, Motooka D, Nishi I, Zin KN, Aye MM, Myint T, Tomono K, Hamada S. 2019. Spreading patterns of NDM-producing *Enterobacteriaceae* in clinical and environmental settings in Yangon, myanmar. Antimicrob Agents Chemother 63:e01924-18. doi:10.1128/AAC.01924-18. 30530602 PMC6395922

[5] Sugawara Y, Akeda Y, Hagiya H, Zin KN, Aye MM, Takeuchi D, Matsumoto Y, Motooka D, Nishi I, Tomono K, Hamada S. 2021. Characterization of *bla*_NDM-5_-harbouring *Klebsiella pneumoniae* sequence type 11 international high-risk clones isolated from clinical samples in yangon general hospital, a tertiary-care hospital in Myanmar. J Med Microbiol 70. https://pubmed.ncbi.nlm.nih.gov/34038339/. 10.1099/jmm.0.001348 34038339

[6] Shen P, Yi M, Fu Y, Ruan Z, Du X, Yu Y, Xie X. 2017. Detection of an *Escherichia coli* sequence type 167 strain with two tandem copies of *bla*_NDM-1_ in the chromosome. J Clin Microbiol 55:199–205. doi:10.1128/JCM.01581-16. 27807154 PMC5228230

[7] Poirel L, Dortet L, Bernabeu S, Nordmann P. 2011. Genetic features of *bla*_NDM-1_-positive *Enterobacteriaceae*. Antimicrob Agents Chemother 55:5403–5407. doi:10.1128/AAC.00585-11. 21859933 PMC3195013

[8] Reynolds ME, Phan HTT, George S, Hubbard ATM, Stoesser N, Maciuca IE, Crook DW, Timofte D. 2019. Occurrence and characterization of *Escherichia coli* ST410 co-harbouring *bla*_NDM-5_, *bla*_CMY-42_ and *bla*_TEM-190_ in a dog from the UK. J Antimicrob Chemother 74:1207–1211. doi:10.1093/jac/dkz017. 30753576

[9] Sakamoto N, Akeda Y, Sugawara Y, Takeuchi D, Motooka D, Yamamoto N, Laolerd W, Santanirand P, Hamada S. 2018. Genomic characterization of carbapenemase-producing *Klebsiella pneumoniae* with chromosomally carried *bla*_NDM-1_. Antimicrob Agents Chemother 62:e01520-18. doi:10.1128/AAC.01520-18. 30323033 PMC6256778

[10] Mathers AJ, Stoesser N, Chai W, Carroll J, Barry K, Cherunvanky A, Sebra R, Kasarskis A, Peto TE, Walker AS, Sifri CD, Crook DW, Sheppard AE. 2017. Chromosomal integration of the *Klebsiella pneumoniae* carbapenemase gene, *bla*_KPC_, in *Klebsiella* species is elusive but not rare. Antimicrob Agents Chemother 61:e01823-16. doi:10.1128/AAC.01823-16. PMC5328509 28031204

[11] He S, Chandler M, Varani AM, Hickman AB, Dekker JP, Dyda F. 2016. Mechanisms of evolution in high-consequence drug resistance plasmids. mBio 7:e01987-16. doi:10.1128/mBio.01987-16. 27923922 PMC5142620

[12] Yang X, Ye L, Li Y, Chan EW, Zhang R, Chen S. 2020. Identification of a chromosomal integrated DNA fragment containing the *rmpA2* and *iucABCDiutA* virulence genes in *Klebsiella pneumoniae*. mSphere 5:e01179-20. doi:10.1128/mSphere.01179-20. 33361128 PMC7763553

[13] Yoon EJ, Gwon B, Liu C, Kim D, Won D, Park SG, Choi JR, Jeong SH. 2020. Beneficial chromosomal integration of the genes for CTX-M extended-spectrum β-lactamase in *Klebsiella pneumoniae* for stable propagation. mSystems 5:e00459-20. doi:10.1128/mSystems.00459-20. 32994286 PMC7527135

[14] Kim TH, Tao X, Moya B, Jiao Y, Basso KB, Zhou J, Lang Y, Sutaria DS, Zavascki AP, Barth AL, Reeve SM, Schweizer HP, Deveson Lucas D, Boyce JD, Bonomo RA, Lee RE, Shin BS, Louie A, Drusano GL, Bulitta JB. 2020. Novel cassette assay to quantify the outer membrane permeability of five beta-lactams simultaneously in carbapenem-resistant *Klebsiella pneumoniae* and *Enterobacter cloacae*. mBio 11:e03189-19. doi:10.1128/mBio.03189-19. 32047131 PMC7018653

[15] Kitchel B, Rasheed JK, Endimiani A, Hujer AM, Anderson KF, Bonomo RA, Patel JB. 2010. Genetic factors associated with elevated carbapenem resistance in KPC-producing *Klebsiella pneumoniae*. Antimicrob Agents Chemother 54:4201–4207. doi:10.1128/AAC.00008-10. 20660684 PMC2944623

[16] Bontron S, Poirel L, Kieffer N, Savov E, Trifonova A, Todorova I, Kueffer G, Nordmann P. 2019. Increased resistance to carbapenems in Proteus mirabilis mediated by amplification of the *bla*_VIM-1_-carrying and IS *26*-associated class 1 integron. Microb Drug Resist 54:4201–4207. 10.1089/mdr.2018.0365 30676261

[17] Yang L, Lin Y, Lu L, Xue M, Ma H, Guo X, Wang K, Li P, Du X, Qi K, Li P, Song H. 2020. Coexistence of two *bla*_NDM-5_ genes carried on IncX3 and IncFII plasmids in an *Escherichia coli* isolate revealed by Illumina and Nanopore sequencing. Front Microbiol 11:195. eCollection 2020. doi:10.3389/fmicb.2020.00195. 32117184 PMC7031209

[18] Dortet L, Nordmann P, Poirel L. 2012. Association of the emerging carbapenemase NDM-1 with a bleomycin resistance protein in *Enterobacteriaceae* and *Acinetobacter baumannii*. Antimicrob Agents Chemother 56:1693–1697. doi:10.1128/AAC.05583-11. 22290943 PMC3318356

[19] Smith MA, Bidochka MJ. 1998. Bacterial fitness and plasmid loss: the importance of culture conditions and plasmid size. Can J Microbiol 44:351–355. doi:10.1139/w98-020. 9674107

[20] Bergstrom CT, Lipsitch M, Levin BR. 2000. Natural selection, infectious transfer and the existence conditions for bacterial plasmids. Genetics 155:1505–1519. doi:10.1093/genetics/155.4.1505. 10924453 PMC1461221

[21] Paul D, Bhattacharjee A, Ingti B, Choudhury NA, Maurya AP, Dhar D, Chakravarty A. 2017. Occurrence of *bla*_NDM-7_ within IncX3-type plasmid of *Escherichia coli* from India. J Infect Chemother 23:206–210. doi:10.1016/j.jiac.2016.12.009. 28131738

[22] González LJ, Bahr G, Nakashige TG, Nolan EM, Bonomo RA, Vila AJ. 2016. Membrane anchoring stabilizes and favors secretion of New Delhi metallo-β-lactamase. Nat Chem Biol 12:516–522. doi:10.1038/nchembio.2083. 27182662 PMC4912412

