# Supplementary material for: Role of Chromosome- and/or Plasmid-Located blaNDM on the Carbapenem Resistance and the Gene Stability in Escherichia coli
Source: Microbiol Spectr. 2022 Aug 2;10(4):e00587-22. doi: 10.1128/spectrum.00587-22 (PMC9430279; doi:10.1128/spectrum.00587-22)
Supplement: Supplemental file 1 — Supplemental material. Download spectrum.00587-22-s0001.pdf, PDF file, 1.2 MB [file spectrum.00587-22-s0001.pdf]

Materials and Methods

Characterization of the bacterial isolate. The clinical isolate of *E. coli* M719 used in this study was obtained from Yangon General Hospital, Yangon, Myanmar, and the WGS data are deposited in the DNA Databank of Japan under the accession numbers APO023433 to APO023437. The location of bla_{NDM} was determined via PFGE of the S1 nuclease- and I-*Ceu*I endonuclease-treated genomic DNA, followed by performing Southern hybridization by targeting bla_{NDM} (1). The DNA fragments digested using S1 nuclease were separated using the CHEF-Mapper XA System (Bio-Rad, Hercules, CA, USA) for 15 h at 6.0 V/cm at 14 °C, with a switch time of 2.98–21.79 s. The DNA fragments were then transferred to a nylon membrane, hybridized with a digoxigenin-labeled probe specific for the bla_{NDM} gene, and detected using CDP-Star® Chemiluminescent Substrate (GE Healthcare Life Sciences, MA, USA). Subsequently, I-*Ceu*I endonuclease PFGE was performed in which the probe was hybridized to the chromosomal position in the experiment described above. DNA fragments digested using I-*Ceu*I endonuclease were separated using the CHEF-Mapper XA System for 12 h at 6.0 V/cm at 14 °C, with a switch time of 20–120 s, followed by a switch time of 60-100 s. Southern blots were probed for the bla_{NDM} and 16S rRNA genes.

WGS and plasmid sequencing. DNA was extracted from *E. coli* M719 using the

PowerSoil DNA Isolation Kit (Qiagen, Hilden, Germany) and then subjected to WGS using the HiSeq 3000 (Illumina, San Diego, CA, USA) and MinION (Oxford Nanopore Technologies, Oxford, United Kingdom) systems to obtain complete chromosome and plasmid sequences. The genomic DNA library for Illumina sequencing was prepared using the KAPA Frag (Kapa Biosystems, Woburn, MA, USA) and TruSeq DNA Nano (Illumina) kits. For MinION sequencing, the library was constructed using the Ligation Sequencing Kit 1D (Oxford Nanopore Technologies). The reads were assembled using Canu (2), cyclized using Circlator (3), and then corrected using Pilon (4). The multilocus sequence typing, ResFinder, and PlasmidFinder databases were used to identify the STs, antimicrobial resistance genes, and plasmid replicon types, respectively (5,6). The sequences were annotated using RASTtk (7), and the genomic structures were compared using EasyFig (8).

Construction of knockout mutants. *E. coli* M719 was used as the WT strain. A small 6.1 kbp plasmid (pYAK1) containing a chloramphenicol resistance gene isolated from an *E. coli* strain (9), was used as a suicide vector for the deletion of bla_{NDM}. *fosA* was subcloned downstream of the Tdh-P promoter in pSA19CP-MCS (10). The *fosA* gene was isolated from *K. pneumoniae* isolate KP64 (11) and amplified using primers *fosA*-F and *fosA*-R containing the *Pst*I and *Xba*I restriction sites of pSA19CP-MCS, respectively. For

primer *fosA*-F, a 15 bp ribosome-binding domain was additionally inserted between the *Xba*I restriction site and the start codon of *fosA*. This *fosA* amplicon was subcloned into the *Pst*I and *Xba*I restriction sites of pSA19CP-MCS using ligation high v2 (Toyobo, Osaka, Japan). pSA19CP-MCS-*fosA* was transformed into *E. coli* DH5α. The fragment containing the Tdh-P promoter and *fosA* gene was extracted from the pSA19CP-MCS derivative using *Eco*RI and *Sma*I. Dephosphorylation and blunting were performed to subclone the fragment into the *Sma*I restriction site of pYAK1. For pYAK1-*fosA*-C, the homologous regions were amplified from two 500 bp fragments upstream and downstream of the $bla_{\text{NDM-5}}$ gene in the chromosome of M719 using primers Frag1-F, Frag1-R, Frag2-F, and Frag2-R. Primers Frag1-F and Frag2-R containing a 15 bp extension homologous to the *Eco*RV and *Nde*I restriction sites of the pYAK1-*fosA* region, and primers Frag1-R and Frag2-F, shared a 15 bp sequence for ligation using the In-Fusion HD Cloning Kit (TaKaRa Bio, Shiga, Japan). pYAK1-*fosA*-P containing a 3800 bp homologous region was constructed in a manner similar to that described above using primers Frag1′-F, Frag1-R, Frag2-F, and Frag2′-R. The deletion mutant $\Delta cbla_{\text{NDM}}$ was constructed by introducing pYAK1-*fosA*-C into the WT strain via bacterial conjugation following 20% sucrose treatment. Mutant $\Delta pbla_{\text{NDM}}$ was constructed in a similar manner using pYAK1-*fosA*-P. A double knockout mutant, $\Delta pcbla_{\text{NDM}}$,was generated using mutant

Δp*bla*$_{NDM}$ by introducing pYAK1-*fosA*-C into the Δp*bla*$_{NDM}$ mutant. Deletion of *bla*$_{NDM}$ was confirmed using polymerase chain reaction (PCR). The scheme of construction of pYAK1-*fosA*-C is summarized in Fig S1. The plasmids and primers used are summarized in Tables S3 and S4, respectively.

Antimicrobial susceptibility testing. We determined the MICs of imipenem and meropenem for WT and mutants Δp*bla*$_{NDM}$, Δc*bla*$_{NDM}$, and Δpc*bla*$_{NDM}$ using the microdilution method described by the CLSI (12). Briefly, we inoculated 5×10^5 colony forming units (CFU)/ml of bacterial suspension into Mueller-Hinton II broth (Becton, Dickinson and Company, Sparks, MD, USA) that was incubated at 35 °C for 18 h. Samples were prepared in triplicate, and each measurement was independently performed five times.

Spectrophotometric analysis of imipenem hydrolysis. The hydrolysis of imipenem was quantitated as previously reported (13). Briefly, 50 µl of imipenem solution or phosphate-buffered saline was mixed with 3.75×10^7 cells/ml of bacterial suspension. The mixture was incubated at 37 °C for 30 min, and the absorbance was measured at 297 nm and 350 nm. Hydrolytic activity was quantitated as described previously. Samples were prepared in triplicate, and each measurement was performed thrice independently.

Quantitative PCR (qPCR). A colony of each sample was inoculated in 3 ml of Luria-

Bertani (LB) medium and incubated at 37 °C overnight. The overnight culture (100 µl) was added to LB medium (5 ml) and cultured at 37 °C until log-phase growth (OD_{600}=0.3). Total RNA was extracted using the NucleoSpin® RNA kit (Qiagen). cDNA was synthesized using ReverTra Ace qPCR RT Master Mix with gDNA Remover (Toyobo). qPCR was performed on a LightCycler® 96 system (Roche Diagnostics, Basel, Switzerland) using SYBR® Green Realtime PCR Master Mix (Toyobo). The mRNA levels of $\textit{bla}_{\mathrm{NDM}}$ were normalized to those of *rpoD*. For determining the plasmid copy number, 1 colony of each sample was inoculated in 3 ml of LB medium and incubated at 37 °C overnight. Total DNA was extracted using a QIAamp DNA Mini Kit (Qiagen) from 1 ml of overnight culture. qPCR was performed as described previously. The plasmid copy number was calculated by normalizing the level of the gene encoding DNA methylase (NCBI Accession No. WP_000085889) located only on pM71901 to the level of *rpoD* (14). Samples were prepared in triplicate, and each measurement was performed thrice independently. The primers used for qPCR are shown in Table S2.

Serial passage assays. The $\textit{bla}_{\mathrm{NDM}}$ deletion mutants $\Delta \mathrm{p}\textit{bla}_{\mathrm{NDM}}$ and $\Delta \mathrm{c}\textit{bla}_{\mathrm{NDM}}$ were subjected to daily passaging as previously described (15). Briefly, three independent lineages were cultured at 37 °C overnight in 5 ml LB medium. Daily passaging of 5 µl of the overnight culture to 5 ml LB medium with and without 0.25 µg/ml meropenem was

performed once a day for 30 days. Subsequently, 50 colonies of each lineage were screened on LB agar plates with and without meropenem to determine the population of *bla*$_{NDM}$-positive cells. The loss of *bla*$_{NDM}$ was confirmed via PCR targeting the region between 1,388 bp upstream and 778 bp downstream of *bla*$_{NDM-5}$. Three independent linages were prepared, and each experiment was performed thrice independently.

References

1. **Liu SL, Hessel A, Sanderson KE**. 1993. Genomic mapping with I-Ceu I, an intron-located endonuclease specific for genes for ribosomal RNA, in *Salmonella* spp., *Escherichia coli*, and other bacteria. Proceedings of the National Academy of Sciences of the United States of America **90**:6874–6878. doi: 10.1073/pnas.90.14.6874.
2. **Koren S, Walenz BP, Berlin K, Miller JR, Bergman NH, Phillippy AM**. 2017. Canu: scalable and accurate long-read assembly via adaptive k-mer weighting and repeat separation. Genome Research **27**:722–736. doi:10.1101/gr.215087.116.
3. **Hunt M, Silva ND, Otto TD, Parkhill J, Keane JA, Harris SR.** 2015. Circlator: automated circularization of genome assemblies using long sequencing reads. Genome Biology **16**:294. doi: 10.1186/s13059-015-0849-0.

4. **Walker BJ, Abeel T, Shea T, Priest M, Abouelliel A, Sakthikumar S, Cuomo CA, Zeng Q, Wortman J, Young SK, Earl AM**. 2014. Pilon: an integrated tool for comprehensive microbial variant detection and genome assembly improvement. PLOS ONE **9**:e112963. doi:10.1371/journal.pone.0112963.

5. **Zankari E, Hasman H, Cosentino S, Vestergaard M, Rasmussen S, Lund O, Aarestrup FM, Larsen MV**. 2012**.** Identification of acquired antimicrobial resistance genes. The Journal of Antimicrobial Chemotherapy **67**:2640–2644. doi:10.1093/jac/dks261.

6. **Carattoli A, Zankari E, García-Fernández A, Voldby Larsen M, Lund O, Villa L, Møller Aarestrup F, Hasman H**. 2014**.** In silico detection and typing of plasmids using PlasmidFinder and plasmid multilocus sequence typing. Antimicrobial Agents and Chemotherapy **58**:3895–3903. doi:10.1128/AAC.02412-14.

7. **Brettin T, Davis JJ, Disz T, Edwards RA, Gerdes S, Olsen GJ, Olson R, Overbeek R, Parrello B, Pusch GD, Shukla M, Thomason JA, Stevens R, Vonstein V, Wattam AR, Xia F**. 2015. RASTtk: a modular and extensible implementation of the RAST algorithm for building custom annotation pipelines and annotating batches of genomes. Scientific Reports **5**:8365. doi:10.1038/srep08365.

8. **Sullivan MJ, Petty NK, Beatson SA**. 2011. Easyfig: a genome comparison visualizer.

Bioinformatics **27**:1009–1010. doi:10.1093/bioinformatics/btr039.

9. **Kodama T, Akeda Y, Kono G, Takahashi A, Imura K, Iida T, Honda T**. 2002. The EspB protein of enterohaemorrhagic Escherichia coli interacts directly with alpha-catenin. Cellular Microbiology **4**:213–222.

10. **Nomura T, Hamashima H, Okamoto K**. 2000. Carboxy terminal region of haemolysin of *Aeromonas sobria* triggers dimerization. Microbial Pathogenesis **28**:25–36. doi: 10.1006/mpat.1999.0321.

11. **Sakamoto N, Akeda Y, Sugawara Y, Takeuchi D, Motooka D, Yamamoto N, Laolerd W, Santanirand P, Hamada S**. 2018. Genomic characterization of carbapenemase-producing *Klebsiella pneumoniae* with chromosomally carried $bla_{\mathrm{NDM-1}}$. Antimicrobial Agents and Chemotherapy **62**:e01520-18. doi: 10.1128/AAC.01520-18.

12. **Abe R, Hagiya H, Akeda Y, Yamamoto N, Ishii Y, Tomono K**. 2019. Bactericidal efficacy of meropenem in combination with cefmetazole against IMP-producing carbapenem-resistant *Enterobacteriaceae*. BMC Research Notes **12**:740. doi: 10.1186/s13104-019-4779-x.

13. **Takeuchi D, Akeda Y, Sugawara Y, Sakamoto N, Yamamoto N, Shanmugakani RK, Ishihara T, Shintani A, Tomono K, Hamada S**. 2018. Establishment of a dual-

wavelength spectrophotometric method for analysing and detecting carbapenemase-producing Enterobacteriaceae. Scientific Reports **8**:15689. doi: 10.1038/s41598-018-33883-0.

14. **Sauer U, Canonaco F, Heri S, Perrenoud A, Fischer E**. 2004. The soluble and membrane-bound transhydrogenases UdhA and PntAB have divergent functions in NADPH metabolism of *Escherichia coli*. The Journal of Biological Chemistry **279**:6613–6619. doi: 10.1074/jbc.M311657200.

15. **Sun J, Chen C, Cui CY, Zhang Y, Liu X, Cui ZH, Ma XY, Feng Y, Fang LX, Lian XL, Zhang RM, Tang YZ, Zhang KX, Liu HM, Zhuang ZH, Zhou SD, Lv JN, Du H, Huang B, Yu FY, Mathema B, Kreiswirth BN, Liao XP, Chen L, Liu YH**. 2019. Plasmid-located *tet* (X). Nature Microbiology **4**:1457–1464. doi: 10.1038/s41564-019-0496-4.

Supplementary Figure legends

FIG S1 Construction of pYAK1-*fosA*-C. Cyan, orange and pink boxes indicate 15-bp extension homologous to the *Eco*RV and *Nde*I restriction sites of the pYAK1-fosA region. Green and light green boxes indicate 500-bp fragments upstream and downstream of the $bla_{\text{NDM-5}}$ gene. Tdh-P Tdh-promoter; Cm^R, chloramphenicol resistant gene; *sacB*, levansucrase-encoding gene, which is lethal to *E. coli* under sucrose treatment.

FIG S2 Genetic environment of the $bla_{\text{NDM-5}}$ gene on the M719 chromosome, plasmid, and the $bla_{\text{NDM-1}}$ gene (18). Alignment of 51 bp promoter sequences were also shown.

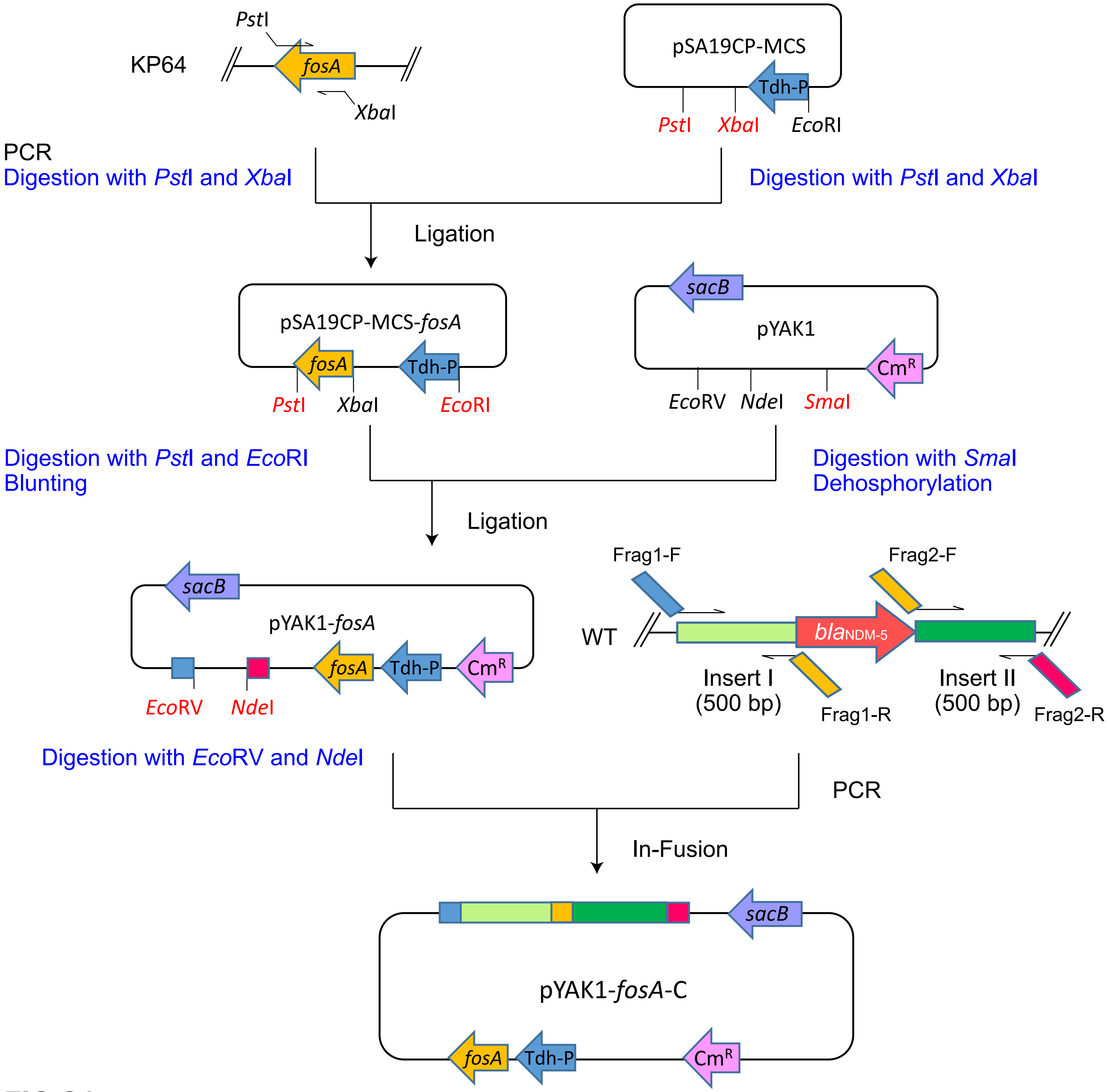
KP64
PstI
fosA
XbaI
pSA19CP-MCS
Tdh-P
PstI
XbaI
EcoRI
PCR
Digestion with PstI and XbaI
Digestion with PstI and XbaI
Ligation
pSA19CP-MCS-fosA
fosA
Tdh-P
PstI
XbaI
EcoRI
sacB
pYAK1
CmR
EcoRV
NdeI
SmaI
Digestion with PstI and EcoRI
Blunting
Digestion with SmaI
Dehosphorylation
Ligation
sacB
pYAK1-fosA
fosA
Tdh-P
CmR
EcoRV
NdeI
Frag1-F
Frag2-F
WT
blaNDM-5
Insert I
(500 bp)
Frag1-R
Insert II
(500 bp)
Frag2-R
Digestion with EcoRV and NdeI
PCR
In-Fusion
sacB
pYAK1-fosA-C
fosA
Tdh-P
CmR

FIG S1

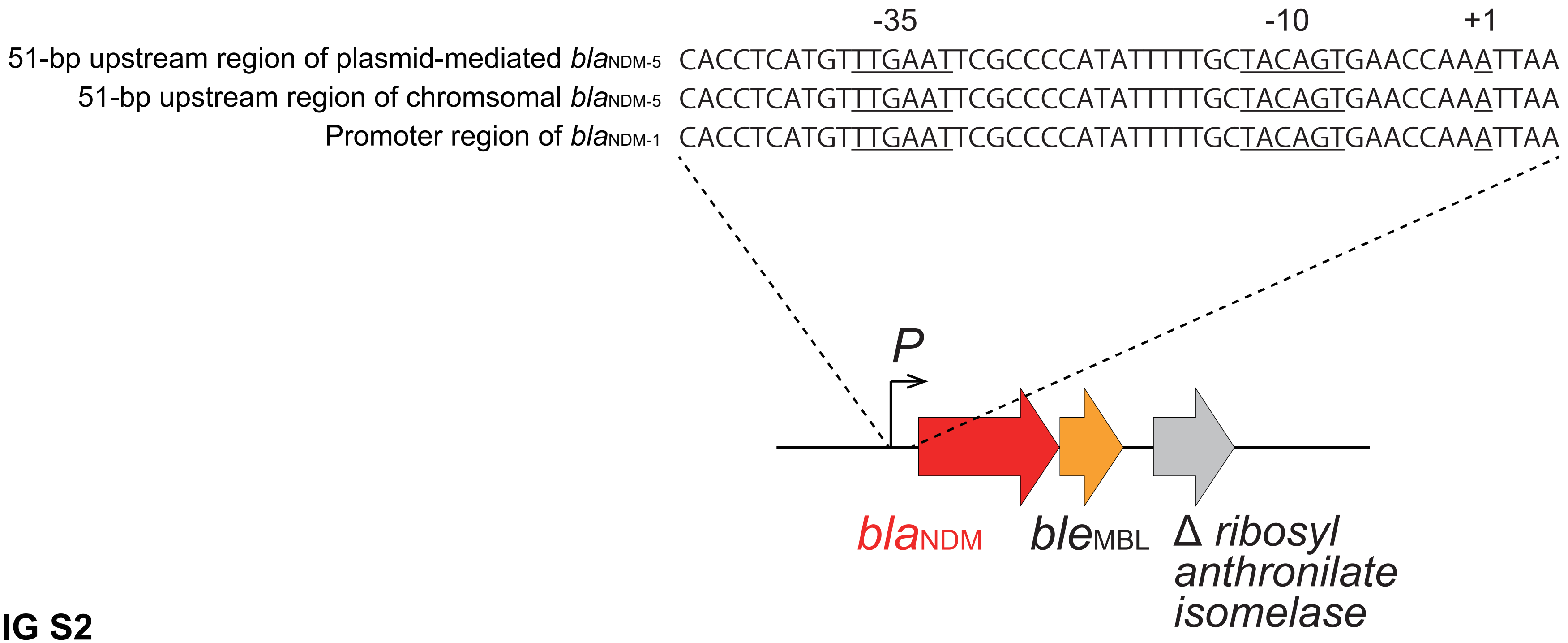

-35
-10
+1
51-bp upstream region of plasmid-mediated blaNDM-5 CACCTCATGTTTGAATTCGCCCCATATTTTTGCTACAGTGAACCAAATTAA
51-bp upstream region of chromsomal blaNDM-5 CACCTCATGTTTGAATTCGCCCCATATTTTTGCTACAGTGAACCAAATTAA
Promoter region of blaNDM-1 CACCTCATGTTTGAATTCGCCCCATATTTTTGCTACAGTGAACCAAATTAA
P
blaNDM
bleMBL
Δ ribosyl anthronilate isomelase

FIG S2

Table S1. Genomic features of *E. coli* isolate M719 containing chromosomal-encoded $bla_{\text{NDM-5}}$ gene

Strain	MLST	Genomic structure	Length (bp)	Resistant genes	Replicon type	Accession No.
M719	8453	Chromosome	4,856,504	*aadA1, aadA2,* $bla_{\text{OXA-1}}$, $bla_{\text{NDM-5}}$, *qepA, catA1, sul1, dfrA12*	-	APO023433
		pM71901	94,605	*aadA2, rmtB,* $bla_{\text{NDM-5}}$, $bla_{\text{TEM1-B}}$, *mph(A), erm(B), sul1, dfrA12*	FII	APO023434
		pM71902	130,897	$bla_{\text{CTX-M-15}}$, $bla_{\text{TEM-1B}}$, *qnr1B, tet(B)*	FIB	APO023435
		pM71903	118,399	-	Y	APO023436
		pM71904	2,287	-	Col	APO023437

Table S2. Stability of the $bla_{\text{NDM-5}}$ gene in *Escherichia coli* M719 mutants over 30 days of serial passaging with and without antibiotic.

Strain	MEPM	% bla_{NDM}-positive cells (±SD)		P value[a]
		Day 0	Day 30	
Δc$bla_{\text{NDM-5}}$	-	100	86.7 (±11.0)	0.0211
Δp$bla_{\text{NDM-5}}$	-	100	100	-
Δc$bla_{\text{NDM-5}}$	+	100	100	-

[a] Significant difference ($p<0.05$) was determined by Student *t* test between % bla_{NDM}-positive cells at day 0 and day 30.

Table S3. Bacterial plasmids used in this study

Plasmid	Description	Resistant Phenotype	Reference
pYAK1	Conjugative suicide vector containing *sacB* gene	Cm^R	10
pSA19-MCS		Cm^R	11
pSA19-MCS-*fosA*	pSA19-MCS derivative containing fosA	Cm^R, FOM^R	This study
pYAK1-*fosA*	pYAK1 derivative containing Tdh-P and *fosA*	Cm^R, FOM^R	This study
pYAK1-*fosA* -C	pYAK1-*fosA* derivative containing 1000-bp homologous region	Cm^R, FOM^R	This study
pYAK1-*fosA* -P	pYAK1-*fosA* derivative containing 3800-bp homologous region	Cm^R, FOM^R	This study

Table S4. Primers used in this study

Target locus	Primer Name	Oligonucleotide sequence (5′ to 3′)
fosA	fosA-F	GGCCTCTAGAACTGGAGGAACAGACATGCTGAG
	fosA-R	CCGGCTGCAGTCACTGATCAAAAAACACCATCCCC
500-bp fragment upstream of *bla*$_{NDM-5}$ gene	Frag1-F	TTTATATAGTTCATAACAGTGCCGTTTACTCATACCT
	Frag1-R	CTAGGGCAATTCCATCAAGTTTTCC
500-bp fragment dowmstream of *bla*$_{NDM-5}$ gene	Frag2-F	ATGGAATTGCCCTAGGACCACGTCACCCCCAATCT
	Frag2-R	AAAGAAAATGCCGATCCCGCGACATTCGACGTAA
1800-bp fragment upstream of *bla*$_{NDM-5}$ gene	Frag1'-F	TTTATATAGTTCATAAGTGCCGAAGAAGGAAGAGC
2000-bp fragment downstream of *bla*$_{NDM-5}$ gene	Frag2'-R	AAAGAAAATGCCGATTCGCCCAGGACTTCGAACT
bla$_{NDM-5}$	NDM-5-F	GCAAATGGAAACTGGCGACC
	NDM-5-R	TCAAACCGTTGGAAGCGACT
rpoD	rpoD-F	GTGATGGAAGAAGCACCGGA
	rpoD-R	GTGCGCCCGATTTCAGATTC
DNA methylase	tra-F	GCGTGTTATGGCCACGATTC
	tra-R	GTTAACGTCACCGGCAATGG
